# Predicting the Receptive Range of Olfactory Receptors

**DOI:** 10.1371/journal.pcbi.0040018

**Published:** 2008-02-01

**Authors:** Rafi Haddad, Liran Carmel, Noam Sobel, David Harel

**Affiliations:** 1 Department of Computer Science and Applied Mathematics, The Weizmann Institute of Science, Rehovot, Israel; 2 Department of Neurobiology, The Weizmann Institute of Science, Rehovot, Israel; University of Auckland, New Zealand

## Abstract

Although the family of genes encoding for olfactory receptors was identified more than 15 years ago, the difficulty of functionally expressing these receptors in an heterologous system has, with only some exceptions, rendered the receptive range of given olfactory receptors largely unknown. Furthermore, even when successfully expressed, the task of probing such a receptor with thousands of odors/ligands remains daunting. Here we provide proof of concept for a solution to this problem. Using computational methods, we tune an electronic nose to the receptive range of an olfactory receptor. We then use this electronic nose to predict the receptors' response to other odorants. Our method can be used to identify the receptive range of olfactory receptors, and can also be applied to other questions involving receptor–ligand interactions in non-olfactory settings.

## Author Summary

A key goal in biology is to identify specific ligands for specific receptors. One example is where the ligand is a drug. In turn, in the olfactory system the ligand is the odorant that binds to olfactory receptors. There are many olfactory receptor types, and which odorants will activate which receptors remains largely unknown. One way to answer this is to systematically vary the molecular features of ligands and to measure the olfactory receptor response. However, the vast number of molecular features and their combinations renders such an effort potentially unsolvable. Here, rather than looking at the trees (each molecular feature), we looked at the forest (the smell they generate). We used a device called an electronic nose that generates a patterned response to odorants. We then obtained the response to a set of odorants that are known to activate a particular olfactory receptor, and we used this pattern to predict the response of that receptor to other odorants. We found that, on average in three out of four we could predict the response of olfactory receptors. This result provides a new method for probing the olfactory system, and also suggests a novel method for identifying potential drugs.

## Introduction

The mammalian sense of smell is able to detect molecules at levels as low as a few parts per trillion, as well as recognize and discriminate thousands of volatile molecules of diverse structure. Smelling begins when airborne odorants traverse the aqueous mucus layer covering the nasal epithelium, and bind to receptor proteins (ORs) within the membrane of cilia stemming from olfactory neurons. These receptors exhibit the characteristic structural features of the super family of G-protein–coupled receptors. In mammals, the repertoire of ORs is extremely large, consisting of over a thousand different subtypes [1–4]. It is currently held that many odorants are recognized by more than one receptor type, and most receptors recognize multiple odorants [5,6]. Olfactory neurons are found in abundance (10–100 million) within the sensory surface, and it is thought that all sensory cells expressing the same receptor type project their axons onto two (or more) topographically fixed glomeruli in the olfactory bulb [7–10]. Therefore, the number of glomeruli is estimated to be between 1,000 and 2,000, namely a reflection of the number of different receptor types. Thus, the *receptive field* of a glomerulus—which is defined as the stimulus range to which it responds—is equivalent to the molecular receptive range of the olfactory receptor expressed by its innervating neurons.

As implied by the above-described organization of the peripheral olfactory system, the initial key to elucidating olfactory coding lies in elucidating the receptive range of given olfactory receptors. Indeed, this goal has been the subject of intensive research in laboratories and computation centres worldwide. Computational methods typically try to estimate the binding affinity based on the structures of the ligand and receptor [11–14]. While unequivocally promising, the complexity of the computations and the paucity of GPCR receptors with known structure put limits on the power of this approach.

In turn, finding the receptive range for a given OR experimentally is difficult because ORs expressed in heterologous cells are typically retained in the endoplasmic reticulum [15]. Furthermore, even when successfully expressed [16–18], the stimulus set size used is rather small compared to the large set of possible stimuli, and therefore capable of covering only a small portion of the receptive ranges.

Here we set out to ask whether we could use artificial olfaction to predict ligand–receptor binding affinity, thereby suggesting a fast and cheap alternative to time-consuming computations or tedious experiments. This possibility has far-reaching consequences for drug design and might find applications in odor communication as well [19].

The most straightforward tools of artificial olfaction are *electronic noses* (eNoses) [20,21]. These are analytic devices that play a constantly growing role as general-purpose odor analyzers. The main component of an eNose is an array of non-specific chemical sensors. An analyte stimulates many of the sensors in the array, and elicits a characteristic response pattern. The sensors inside the eNose are made using diverse technologies, but in all cases a certain physical property is measured and a set of signals is generated. Electronic noses have been used intensively for many applications, mostly for classification tasks, with considerable success. For example, eNoses have been used in medical applications [22–25], for environmental control [26], for quality assessment of food products [27–31], in the car manufacturing industry [32], in predicting biological activity of alcohols [33], and even in predicting human percept [34].

The results of these applications imply that a good eNose captures enough information on an odorant to allow its discrimination from other odorants [35]. A considerable part of this information is likely to overlap with the factors that determine the interaction strength with the biological receptor, such as relevant structural motifs, surface physical shape, and charge distribution [21]. Consequently, we hypothesized that eNose fingerprints capture information that is relevant to the strength of the biological interaction.

In the first systematic study of OR receptive range, Araneda et al. [16] measured the response of the rat I7 OR to 90 pure chemicals, and divided the results into four groups according to the level of response to the odorant stimulus: high, medium, low, and no response. In a later and more comprehensive effort, Hallem et al. [36] measured the response of 24 Drosophila ORs to a set of 110 odorants. Here we set out to ask whether we could tune an eNose to these results such that the eNose could then predict the receptor response.

## Results

To test this hypothesis, we took it upon ourselves to measure the set of chemicals from the work of Araneda et al. [16] with the MOSESII eNose [37], and to test how well the resulting digital fingerprints can serve as predictors of the interaction strengths with the I7 receptor. For various reasons (e.g., no CAS identification in Araneda et al.), we were able to obtain only 39 of the original 90 chemicals used (see Methods). Out of these 39 chemicals, 28 generated low response or did not respond at all, whereas the remaining 11 generated medium or high response.

**Figure 1:**
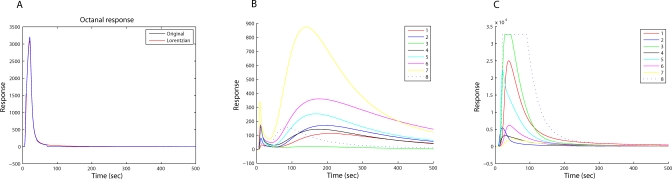
An Electronic Nose Possible Signals (A) A typical signal (Octanal, measured with a QMB sensor; solid red) and the best fitting Lorentzian model (dashed blue). (B) Abnormal signals with double peak. (C) Abnormal signals with out-of-range signals.

A typical eNose signal consists of a few hundred measured values per sensor, thus giving rise to a rather large dataset (Figure 1), necessitating a preliminary stage of feature extraction in the data analysis pipeline. The most popular feature extraction techniques in the field of eNoses capture only a small portion of the information contained in the signals [38]. While such partial methods are satisfactory for many applications, the limited size of the current dataset necessitated maximization of the amount of captured information. To this end, we used the Lorentzian technique, a recently developed method for feature extraction that is particularly suited for this task [38]. This technique is based on fitting the measured signal to an analytic curve (Figure 1), developed using simple assumptions regarding the measurement system and the interaction between an analyte and the sensors. The Lorentzian technique uses four parameters to characterize each signal curve. These four parameters can be used to reconstruct the original signal with high accuracy.

A typical response pattern elicited in a single sensor is shown in Figure 1A. However, part of the 39 chemicals used in this work displayed abnormal response patterns involving multiple-peaks or partial corruptions (Figure 1B and 1C). While all the known feature extraction techniques fail for such abnormal signals, we were able to generalize the Lorentzian technique so that it could be used for any kind of signal in our dataset [39,40].

The Lorentzian technique uses four parameters to characterize each signal. Combined with the 16 sensors of the eNose, this generates a 64-dimensional digital fingerprint. We measured ten repetitions per odorant, totalling 390 samples. Typically, the first sample of each batch somewhat deviated from the rest of the samples (a phenomenon known as conditioning), and it was removed from further analysis. After removing some additional outliers (using Euclidian distance, the samples that were more than two standard deviation away from the mean sample), we were left with a total of 342 samples, 8–9 per odorant.

**Figure 2:**
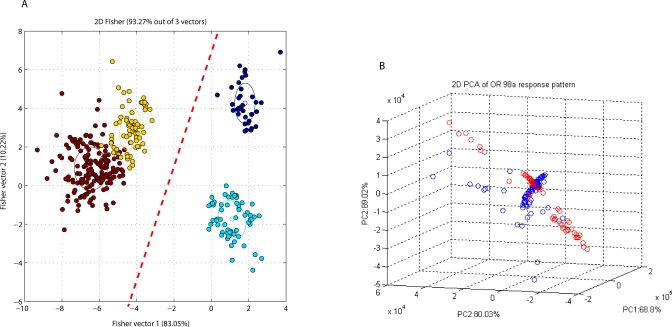
Projecting the Samples into Lower Space (A) A two-dimensional plot of the eNose digital fingerprints. The raw data were transformed using the Fisher transformation, which maximizes class separability, defined as the ratio of the between-class scatter matrix to the within-class scatter matrix. A suggested separating hyper-plane between the weak and no-response groups to the groups of medium and high response gives 99.4% correct classification on the training set. Here, non-responding samples are brown, low-responding ones are yellow, medium-responding ones are cyan, and strong-responding ones are blue. (B) A three-dimensional sample plot of OR 98a eNose digital fingerprint from the *Drosophila* experiment using PCA. Here, weak or non-responding are red, and medium or strong response are blue.

Due to the relatively small size of our dataset, we used a coarser two-class classification scheme, according to whether the response was significant, e.g., medium or high, versus whether there was no response at all or a low response. In Figure 2 we visualize this dataset in two dimensions using the Fisher transformation [41]. This transformation is a linear transformation that finds new linear combinations of the original features that optimally discriminate between the different classes.

We then applied a classification algorithm known as the *perceptron* [42]. To calculate the success rate of our prediction rule, we used a leave-one-group-out procedure in the following way: we repeatedly removed one group of samples of the same odorant from our dataset, to form the test set. The remaining samples formed the training set. We then trained a perceptron classifier, while transforming the training data with the Fisher transformation. The success rate is than defined as the average of the 39 leave-one-group-out average runs. The success rate obtained was 76.1% (χ^2^ test, *p* = 0.019). In other words, using an eNose, we were able to predict the I7 receptor response to an odorant in 76% of cases.

### Generalizing to Other Olfactory Receptors

Encouraged by the prediction accuracy for the activity of the rat I7 receptor from only a small subset of chemicals, we set out to further test this approach on more OR data. A recent study measured the response of 24 Drosophila ORs to a set of 110 odorants [36]. We therefore took upon ourselves to measure these chemicals using the same eNose.

We first measured 70 chemicals from [36] using the MOSESII eNose. Each sample was measured 3–4 times. Since in some cases the chemicals measured did not elicit a strong response, we modified the eNose measurement technique by increasing the temperature and the flow rate (see Methods). This insured that almost all chemicals elicited a clear response. We than asked if we could predict the activity of each of the 24 receptors from our eNose signals.

To find a statistically good learning rule that can predict the correct class of an unknown sample, the training set must contain a substantial number of examples from each class. Out of the 24 ORs measured, 12 responded to less than 15% of the odorants (for example, OR 2a responded weekly to only three odorants). We therefore ignored these receptors and did not try to develop a learning rule for them. To find the learning rule for the remaining 12 Ors, we applied the perceptron learning scheme again. We grouped the odorants into two classes. The first contained all the odorants that elicited a weak response or no response (less than 50 spikes, as defined in Hallem et al.), and the second class contained all odorants that had a medium or high response (above or equal 50). Thanks to the larger dataset, we could now use simpler feature extraction methods compared to those we used for the I7 receptor. Each sample was represented by a vector of size 120. This vector contained four datapoints from each of the 16 signals (see Methods), and 56 values that represent the 28 ratios between the maximum values of the eight signals from the metal-oxide (MOX) sensors, and 28 ratios of the eight signals from the quartz microbalance (QMB) sensors. Presenting a vector of size 120 to a neural network increases its computation time and might reduce the success rate. We thus applied PCA and took the eigenvectors that covered 99.9% of the variance. This reduced the size of the input vector by a factor of 2/3.

**Figure 3:**
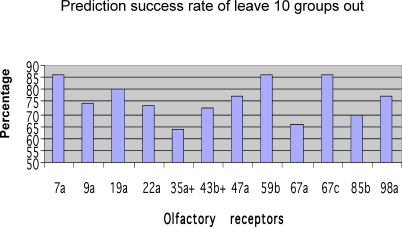
The Prediction Success Rates of the 12 ORs We Tested Using Leave-10-Groups-Out Process The average success rate across all receptors was 77.5%. The success rates of the ORs marked with “+” did not differ significantly from the null hypothesis prediction rule (χ^2^ test , *p* = <0.01).

**Figure 4:**
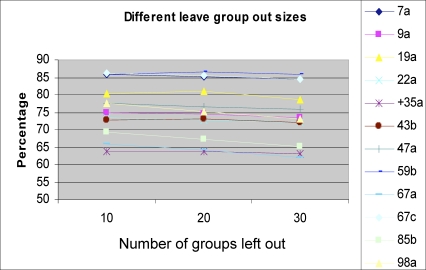
The Prediction Success Rates of the 12 ORs When Using Different Group Sizes as Test Set

To test our learning rule, we applied a “leave 10 groups out” procedure. We performed each test 500 times. On each test, we randomly removed ten odorants and all their repeated measurements; this set was defined as the test set. The remaining odorants were used as a training set. We then trained a perceptron. The success rate for each receptor was defined as the average success rate of all the 500 runs. The results are depicted in Figure 3. As can be seen, in almost all the receptors we tested the success rates were above 70%, with the best success rate being 86% for receptor ORs 7a, 59b, and 67c, and the lowest being 63% for receptor OR 35a. We tested the significance of the result using a χ^2^ test (by comparing the expected 2-classes distribution of the null hypothesis to the observed 2-classes distribution of the learned rule. The null hypothesis is to always predict the class most abundant). We also tested the significance by using a 2-samples *t*-test to compare the mean success rate of the null hypothesis versus the learned rule. As can be seen in Figure 3, in ten out of 12 receptors we tested, the predictive power was significant (χ^2^ test, *p* < 0.01). To further test the strength of this method, we applied a leave group out test of various group sizes. As seen Figure 4, the prediction rates slightly diminish when larger group sizes were used.

To further verify that the results cannot be attributed to chance, we tested the ability to predict a randomly created OR response. To do this, we randomly shuffled the response vector of each OR and then tested if we can learn the pseudo created OR response using the same algorithm. We repeated this randomization process 30 times for each receptor and calculated the average prediction success rate for the 12 randomly created receptors, which turned out to be 56%, a value not different from chance (T(22) = −1.04, *p* = 0.31) and significantly below our results with real OR responses (T(22) = −6.05, *p* = 0.0001).

### Testing the Prediction Rule on New Data

**Figure 5:**
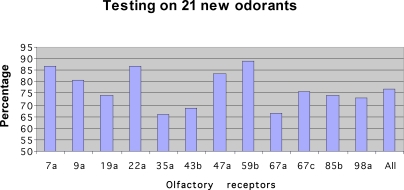
The Result of Predicting the Response of 21 New Odorants Using the Prediction Rules Developed from the Set of 70 Odorants

The most convincing way to test such empirically developed rules is to use data that was not used in the rule building set. To this end, we set out to test an additional 21 odorants that were tested in [36], but were not part of the 70 odorants used above. We measured each of these 21 odorants 3–4 times using our eNose, with the same parameters and outlier removal scheme as used above. This process generated 54 samples, representing 2–3 measurements of each of the 21 odorants. We then checked the prediction success rates of our previously learned rules on these new odorants. The results are depicted in Figure 5. The average prediction rate was 77.07%, where the maximum was 88.8%. In 3/4 of the ORs, we obtained a success rate of above 70%, while in three ORs we obtained a success rate of ∼64% (ORs 67a, 43b, and 35a). The prediction values of a few sample ORs of the list of 21 odorants is detailed in Table 1. In other words, we were able to predict the response to odorants that were not used in our original model building set.

**Table 1:**
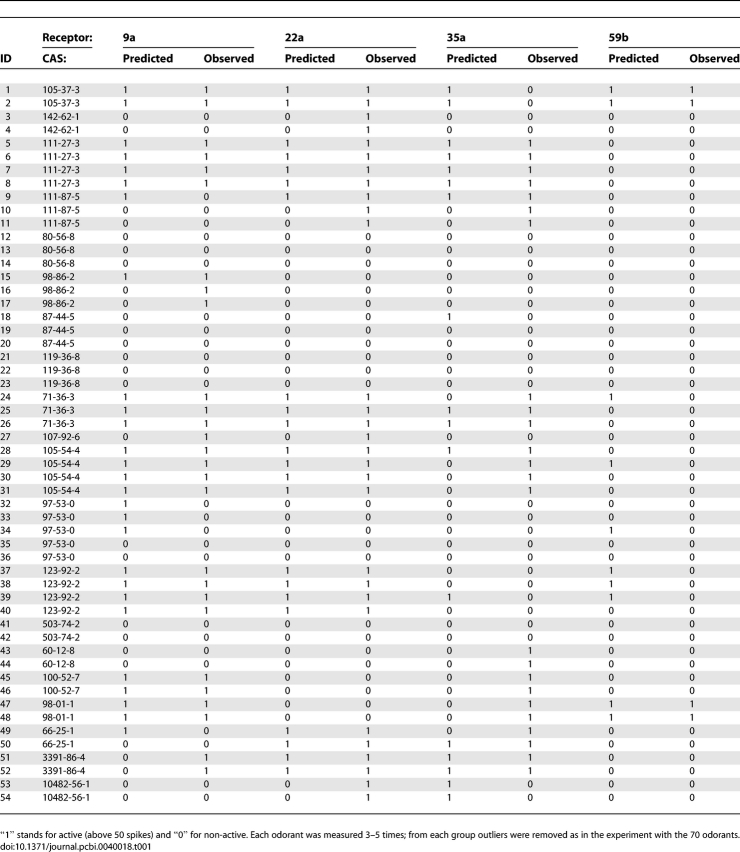
A Few Examples of Prediction Values versus the Observed Values on the Set of the 21 New Odorants

## Discussion

Predicting the response of an OR from the molecular features of an odorant ligand has met limited success. As the number of molecular features involved in the receptor-ligand interaction can be very high and the number of subsets and relations between these features grows very fast, the task of identifying the important features and relations is daunting. With this in mind, here we quantified the response to an odorant in synthetic sensors, in order to obtain an indirect measure of the forces and parameters involved.

Our results suggest that an eNose can be used to predict, with reasonable accuracy, the response of an OR to an unknown odorant. This provides a link between artificial and biological olfaction. The fact that we could not duplicate the successful learning process for the pseudo-receptors lends further credence to this link. The observation that eNoses capture information that is relevant to biological interaction is promising in that they can provide a fast path for analysing the relationship between odorant and receptor, and can be extended to other kinds of biological interaction, outside the realm of olfaction.

The prediction rates for 21 new samples using the 12 ORs tested ranged from 64% to 89%. One may ask what underlies this variability in classification power. Because we could not explain these differences from the receptor response characteristics or the learning scheme (we tried several other learning methods, but in general the results were similar), we hypothesize that one source of the differences might be the noise in the measurements—of both the olfactory response and the eNose. Another likely source is that the ORs for which we could find only weak classifiers are tuned to molecular features that are not captured by the current eNose technology. This implies that an eNose with a greater number of sensor modules will provide more information and will improve the ability to extract more accurate rules. Finally, in this respect, it is noteworthy that even when the prediction rates are not high (but significantly differ from the null hypothesis rule) they can be further improved using boosting methods (from M. Kearns, Thoughts on hypothesis boosting, unpublished manuscript, December 1988), that turn a collection of weak classifiers into a single, highly accurate, classifier. Such boosting could also be achieved by using 2–3 independent eNoses.

One may argue that our solution provides a sort of “black box”: we can tell which odorants may elicit a receptor response, but we can't say why. In other words, we have not directly generated insight regarding the molecular features dominating the response of a particular receptor. We would argue, however, that our results remain valuable even in the absence of mechanistic understanding: experimentally, such predictions may significantly contribute to the study of the olfactory system by providing a path to stimulus selection. If one wants to drive the olfactory system in order to probe its function systematically, one can use this method to judiciously select stimuli. Outside of the experimental context, this method may pave a unique path to ligand identification within a clinical framework. If one has in hand a particular target ligand, one can now use this method to identify additional potential ligands. Although one may indeed remain in the dark as to *why* these ligands are effective, one would nevertheless have potential ligands in hand. Finally, and critically in this respect, a key advantage in our approach is that the use of an eNose allows the application of the method to mixtures of odorants, which is not feasible for methods that quantify the response by recognizing the individual molecular features.

All that said, this method may also set a path toward more mechanistic understanding as well. Such understanding may be achieved by in-depth examination of the particular sensors that dominate the response in one case or another, or by systematic examination of ligands that are grouped by this method, asking what molecular features are common to these ligands. Although such ligand groupings could be equally obtained by targeting odorants at expressed biological receptors, using an eNose for this task is orders-of-magnitude easier, faster, and cheaper.

## Materials and Methods

### The I7 OR experiment.

The list of the 39 pure chemicals we used, and their interaction strength with the I7 rat OR, appear in Table 2.

**Table 2:**
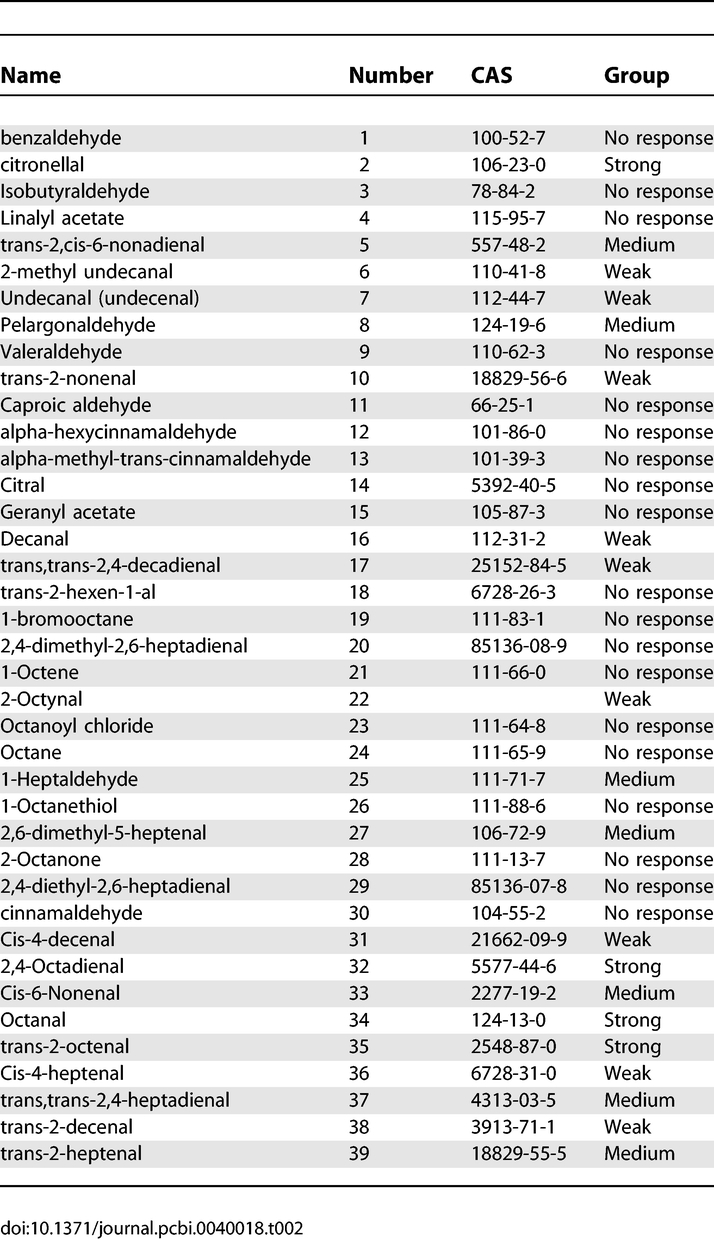
A List of the 39 Chemicals We Used for Our Measurement and Their Response Level in the I7 Experiment

Out of the 90 chemicals used in the I7 experiments, only 14 had a high or a medium response. We managed to measure 11 of them (some of them could not be measured by the eNose). From the remaining low responding or non-responding 76 chemicals, we were able to obtain 28. These numbers constitute a good representation of both response groups. We were unable to obtain the additional odorants because they were not identified by CAS, and were thus of ambiguous identity.

### The *Drosophila* ORs experiment.

We used 91 out of the 110 chemicals used in the *Drosophila* ORs experiments (various technical issues such as low boiling temperature prevented us from obtaining or measuring the remaining 19 odorants). The list of the 91 pure chemicals used appears in Table 3.

**Table 3:**
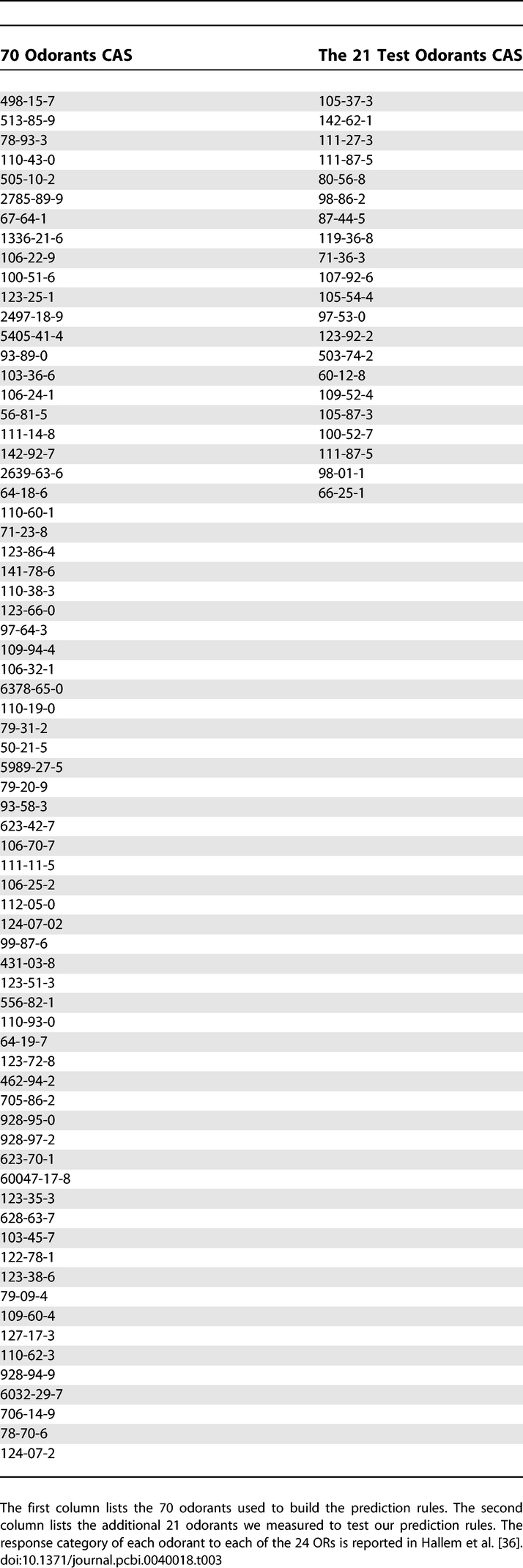
The List of Odorants Used in the 24 *Drosophila* ORs Experiment

### eNose.

The MOSES II eNose we used contains eight metal-oxide (MOX) sensors and eight quartz microbalance (QMB) sensors. MOX and QMB are two very different sensor technologies that together capture many facets of the ligand's nature.

In the I7 experiments, the samples were put in 20-ml vials in an HP7694 headspace sampler, which heated them to 40 °C and injected the headspace content into the MOSES II with a flow rate of 25 ml/litter. There, each analyte was first introduced into the QMB chamber, whence it flowed through to the 300 °C heated MOX chamber. The injection lasted 30 s, and was followed by a 15 min purging stage using synthetic air. Each chemical was measured in batches, with a single batch containing ten successive measurements. In total, we performed 390 measurements. Each odorant was measured at the same level of humidity and temperature. Each single measurement consisted of 16 time-dependent signals, corresponding to the 16 sensors.

In the *Drosophila* 24 ORs experiment, we heated the oven to 50 °C and increased the flow to 40 ml/litter. These parameter changes increased the number of chemicals that elicit a strong response. To avoid the problem of conditioning, we put a blank vial before every measurement and we cleaned the system using steamed air after each run. We measured each sample 3–4 times.

### Feature extraction methods.

In the first experiment, we used the Lorentzian method. Although this method outperforms simple methods commonly used in eNose applications, the Lorenzian method is, however, a lengthy process in which all abnormal signals need to be fixed, as described in [39,40]. Due to the large number of odorants used in the *Drosophila* experiment we decide to use a different feature extraction method that is similar to the Lorentzian but is easier to apply to all kind of signals. This method extracts four parameters from each signal. These parameters are: the signal max value and the time it reaches it, the time the signal reaches the half max value on the decay part and on the rise part. These four parameters are similar to the four parameters used in the Lorentzian model. In many cases the signal max value can change considerably between measurements of the same odorants; however, the relative height of the eight sensors is kept. Thus, to capture this behaviour we added to each odorant representation the 28 possible ratios of the eight MOX signals and 28 ratios of the eight QMB signals. We thus ended up with 120 features for each odorant. To ask whether this feature extractions method is a good representation of the odorants, we clustered the 273 eNose measurements we had into 70 classes and tested how many odorants fall into other classes. Out of the 273 measurements, 85% clustered either to their class or to the class closest to their class, suggesting that this feature extraction method is a good representation of the odorants.

### Perceptron.

We used the Matlab [43] implementation of perceptron. We presented the training set to the perceptron and calculated the training error. Since perceptron is guaranty to converge only for linearly separable problems, we stopped the training if there was no improvement in three consecutive epochs, or when we reached 100 epochs.
